# QuEChERS-超高效液相色谱-串联质谱分析鱼中13种全氟及多氟烷基化合物

**DOI:** 10.3724/SP.J.1123.2023.08002

**Published:** 2024-08-08

**Authors:** Xiaoqi LIU, Zhenzhen LIU, Meiyu WANG, Chenshu GU, Xinquan WANG, Lianliang LIU, Peipei QI

**Affiliations:** 1.宁波大学食品与药学学院, 省部共建农产品质量安全危害因子与风险防控国家重点实验室, 浙江 宁波 315832; 1. State Key Laboratory for Managing Biotic and Chemical Threats to the Quality and Safety of Agro-products, School of Food and Pharmacy, Ningbo University, Ningbo 315832, China; 2.浙江省农业科学院农产品质量安全与营养研究所, 省部共建农产品质量安全危害因子与风险防控国家重点实验室, 浙江 杭州 310022; 2. State Key Laboratory for Managing Biotic and Chemical Threats to the Quality and Safety of Agro-products, Institute of Agro-product Quality, Safety and Nutrition, Zhejiang Academy of Agricultural Sciences, Hangzhou 310022, China

**Keywords:** 磁性吸附剂, 超高效液相色谱-串联质谱, 全氟及多氟烷基化合物, 鱼, magnetic adsorbent, ultra-high performance liquid chromatography-tandem mass spectrometry (UHPLC-MS/MS), perfluorinated and polyfluoroalkyl substances (PFASs), fishes

## Abstract

全氟及多氟烷基化合物(PFASs)广泛存在于全球环境水体中,鱼类等水产品的摄入可能是人类接触PFASs的重要来源,因此亟需建立鱼类产品中PFASs的高效、准确分析技术。本研究以磁性纳米材料为净化吸附剂,基于QuEChERS方法,建立了鱼类产品中13种PFASs的超高效液相色谱-串联质谱(UHPLC-MS/MS)分析方法。实验分别考察了萃取溶剂类型、净化吸附剂(Fe_3_O_4_-TiO_2_和*N*-丙基乙二胺(PSA))用量对PFASs回收率的影响,确定了最佳样品前处理条件。采用Luna Omega C18色谱柱(100 mm×2.1 mm, 1.6 μm)进行分离,利用电喷雾电离(ESI)源,在多反应监测(MRM)模式下采集质谱数据,内标法定量。在优化的实验条件下,13种PFASs在0.01~50 μg/L内具有良好的线性关系,相关系数(*R*)≥0.9973,检出限为0.001~0.023 μg/L,定量限为0.003~0.078 μg/L。在低、中、高3个加标水平(0.5、10、100 μg/kg)下,13种PFASs的加标回收率为78.1%~118%,日内精密度为0.2%~11.1%,日间精密度为0.8%~8.7%。将该方法应用于实际样品分析,所有鱼样品中均有PFASs检出,分别为全氟辛酸、全氟辛基磺酸、全氟十一烷酸、全氟十二烷酸和全氟十三烷酸,各自的检出总含量为0.327~1.728 μg/kg。本方法前处理过程简单且灵敏度高,适用于鱼类产品中PFASs的分析。

## Abstract (en)

Perfluorinated and polyfluoroalkyl substances (PFASs) are compounds characterized by at least one perfluorinated carbon atom in an alkyl chain linked to side-chain groups. Owing to their unique chemical properties, these compounds are widely used in industrial production and daily life. However, owing to anthropogenic activities, sewage discharge, surface runoff, and atmospheric deposition, PFASs have gradually infiltrated the environment and aquatic resources. With their gradual accumulation in environmental waters, PFASs have been detected in fishes and several fish-feeding species, suggesting that they are bioconcentrated and even amplified in aquatic organisms. PFASs exhibit high intestinal absorption efficiencies, and they bioaccumulate at higher trophic levels in the food chain. They can be bioconcentrated in the human body via food (e. g., fish) and thus threaten human health. Therefore, establishing an efficient analytical technique for use in analyzing PFASs in typical fish samples and providing technical support for the safety regulation and risk assessment of fish products is necessary. In this study, by combining solvent extraction and magnetic dispersion-solid phase extraction (d-SPE), an improved QuEChERS method with ultra-high performance liquid chromatography-tandem mass spectrometry (UHPLC-MS/MS) was developed for the determination of 13 PFASs in fish samples. Fe_3_O_4_-TiO_2_ can be used as an ideal adsorbent in the removal of sample matrix interference and a separation medium for the rapid encapsulation of other solids to be isolated from the solution. Based on the matrix characteristics of the fish products and structural properties of the target PFASs, Fe_3_O_4_-TiO_2_ and *N*-propyl ethylenediamine (PSA) were employed as adsorbents in dispersive purification. The internal standard method was used in the quantitative analyses of the PFASs. To optimize the sample pretreatment conditions of analyzing PFASs, the selection of the extraction solvent and amounts of Fe_3_O_4_-TiO_2_ and PSA were optimized. Several PFASs contain acidic groups that are non-dissociated in acidic environments, thus favoring their entry into the organic phase. In addition, acidified acetonitrile can denature and precipitate the proteins within the sample matrix, facilitating their removal. Finally, 2% formic acid acetonitrile was used as the extraction solvent, and 20 mg Fe_3_O_4_-TiO_2_, 20 mg PSA and 120 mg anhydrous MgSO_4_ were used as purification adsorbents. Under the optimized conditions, the developed method exhibited an excellent linearity (*R*≥0.9973) in the range of 0.01-50 μg/L, and the limits of detection (LODs) and quantification (LOQs) ranged from 0.001-0.023 and 0.003-0.078 μg/L, respectively. The recoveries of the 13 PFASs at low, medium, and high spiked levels (0.5, 10, and 100 μg/kg) were 78.1%-118%, with the intra- and inter-day precisions of 0.2%-11.1% and 0.8%-8.7%, respectively. This method was applied in analyzing real samples, and PFASs including perfluorooctanesulfonic acid, perfluorooctanoic acid, perfluoroundecanoic acid, perfluorododecanoic acid, and perfluorotridecanoic acid, were detected in all 11 samples evaluated. This method is simple, sensitive, and suitable for use in analyzing PFASs in fish samples.

全氟及多氟烷基化合物(perfluorinated and polyfluoroalkyl substances, PFASs)是至少含有一个全氟化碳原子的有机化合物,根据其侧链基团种类,PFASs可分为全氟烷基羧酸类、全氟烷基磺酸类、全氟烷基磺酰胺类等^[1]^。PFASs中C-F键的键能极高,具有强疏水性和强疏油性,PFASs已被广泛应用于户外用品、家具及纺织品等产品制造中^[2,3]^。PFASs具有持久性、生物累积性及长距离迁移性^[4]^,可通过人为活动、大气沉降、地表径流、淋溶作用等途径在环境介质间迁移和转化^[5]^。研究表明,PFASs广泛存在于全球环境水体中^[6]^,在韩国^[7]^、越南^[8]^、中国^[9]^,甚至在一些人迹罕至的地区^[10]^均有PFASs的检出。同时,PFASs能够在食物链的更高营养级中产生生物累积,水体中的PFASs会通过生物富集进入到鱼类等水生生物中,再进一步通过食物链富集于人体内,从而对人体健康造成威胁^[11]^。研究表明,鱼类等水产品的摄入可能是人类接触PFASs的重要来源^[12]^,因此亟需建立针对鱼类产品中PFASs的高效分析方法。

鱼类产品富含蛋白质、脂肪和无机盐,而PFASs与蛋白质之间的高亲和力使得样品中目标物与蛋白质、脂肪等基体杂质之间的有效分离面临挑战。目前针对鱼类产品中PFASs的前处理,主要通过将溶剂萃取与固相萃取(SPE)^[13]^、分散固相萃取(d-SPE)^[12]^等净化技术相结合的方式来实现,其中溶剂萃取与d-SPE结合的QuEChERS方法因具有快速、便捷、高性价比等优势而得以广泛应用。传统的QuEChERS方法在溶剂萃取和d-SPE两个净化环节中均需采用高速离心操作,以实现目标物溶液与固体基质、净化吸附剂的分离,但这对于大量样品的高通量分析来说仍存在挑战。磁性吸附材料因具有吸附性好、易分离等优势,在快速样品前处理技术领域受到了广泛关注。在d-SPE过程中,将磁性功能材料作为净化吸附剂,可以快速完成基质与目标物溶液的分离^[14⇓⇓-17]^。Liu等^[18]^将磁性聚合物材料(Poly-MDN)作为净化吸附剂,构建了水产品中的农药残留分析技术,该方法的前处理过程简单、快速,分离效果好且灵敏度高。目前尚未见将磁性功能材料作为d-SPE吸附剂用于鱼类产品中PFASs分析的相关研究报道。

Fe_3_O_4_磁性纳米颗粒因比表面积大、生物相容性高、毒性低和可回收等优点而受到广泛关注,利用溶剂热法所合成出的Fe_3_O_4_磁性纳米颗粒表面存在的大量羟基可作为改性剂的耦合位点^[19]^。TiO_2_纳米颗粒具有较大的比表面积和介孔结构,其结构中的Ti-O键可以使TiO_2_纳米颗粒表面的水分子得到极化,从而与基质中的蛋白质、碳水化合物、脂类等产生氢键作用^[20]^。通过在Fe_3_O_4_纳米颗粒表面修饰TiO_2_,可得到复合磁性吸附剂Fe_3_O_4_-TiO_2_;将Fe_3_O_4_-TiO_2_引入复杂基质中,蛋白质等基质会通过氢键相互作用吸附到Fe_3_O_4_-TiO_2_表面。据此,基于Fe_3_O_4_-TiO_2_的高吸附性能,可实现鱼类样品中复杂基质的净化^[21]^;同时,Fe_3_O_4_-TiO_2_具有超顺磁性,可作为分离介质用于固相与液相的快速分离。

基于上述问题,本研究针对鱼类产品的基质特点和PFASs的结构特点,利用超声辅助有机溶剂萃取和磁性d-SPE技术,结合QuEChERS-超高效液相色谱-串联质谱(UHPLC-MS/MS),构建了鱼类产品中13种PFASs的分析方法,为鱼类产品中PFASs的安全监测和控制提供了技术支撑。

## 1 实验部分

### 1.1 仪器、试剂与材料

Nexera X2 LC-30AD超高效液相色谱仪,配备Shimadzu8050三重四极杆质谱仪、氮气发生器(日本岛津公司); BSA224S分析天平(北京赛多利科学仪器有限公司); KQ-300DE型数控超声波清洗器(昆山市超声仪器有限公司); VX-Ⅲ多管涡旋振荡器(北京踏锦科技有限公司); Milli-Q超纯水仪(美国Millipore公司)。

13种标准品:全氟丁基磺酸(PFBS,纯度99.4%)、全氟己基磺酸(PFHxS,纯度95.0%)、全氟庚基磺酸(PFHpS,纯度96.2%)、全氟辛基磺酸(PFOS,纯度95.0%)、全氟癸基磺酸(PFDS,纯度95.0%)、全氟庚酸(PFHpA,纯度97.4%)、全氟辛酸(PFOA,纯度97.6%)、全氟癸酸(PFDA,纯度96.5%)、全氟十一烷酸(PFUnDA,纯度98.4%)、全氟十二烷酸(PFDoDA,纯度95.3%)、全氟十三烷酸(PFTrDA,纯度98.0%)、全氟己酸(PFHxA,纯度98%)、1-氯代烷基醚磺酸(F-53B,纯度99.9%)均购自天津阿尔塔科技有限公司;两种内标:^13^C_2_-全氟癸酸(^13^C_2_-PFDA,纯度>98.0%)、^13^C_4_-全氟辛烷磺酸钠(^13^C_4_-PFOS,纯度>98.0%)购自加拿大Wellington Laboratories公司;PSA购自Agela Technologies公司(中国上海);无水MgSO_4_、NaCl(分析级)购自中国凌峰化学试剂有限公司;甲醇、乙腈(HPLC级)购自美国Merck公司;实验用水均为Milli-Q超纯水仪所制备的超纯水。聚醚砜(PES)膜(孔径0.22 μm,直径13 mm)购自成都摩尔公司;Fe_3_O_4_-TiO_2_为实验室自制,具体制备过程见参考文献[19]。所有鱼类产品均采自浙江省水产养殖基地。

### 1.2 溶液的配制

单标储备液和内标储备液:分别移取13种标准品(10 mg)于50 mL容量瓶中,用甲醇溶解并定容,配制成质量浓度为200 mg/L的单标储备液,于4 ℃条件下储存;分别移取2种内标(各0.1 g)于10 mL容量瓶中,用甲醇定容,配制成质量浓度为10 mg/L的内标储备液,于4 ℃条件下储存。

混合标准工作溶液和内标混合工作溶液:分别准确移取0.5 mL单标储备液于10 mL容量瓶中,混合均匀后用甲醇定容,配制成质量浓度为10 mg/L的混合标准工作溶液;分别准确移取1 mL内标储备液于10 mL容量瓶中,用甲醇定容,配制成质量浓度为1 mg/L的内标混合工作溶液。

溶剂混合标准溶液和基质匹配混合标准溶液:移取适量混合标准工作溶液,用甲醇进行逐级稀释,再分别加入10 μL内标混合工作溶液,配制成系列质量浓度(0.01、0.02、0.05、0.1、0.2、0.5、1、2、5、10、20、50 μg/L)的溶剂混合标准溶液;移取适量混合标准工作溶液,用空白样品提取液进行逐级稀释,再分别加入10 μL内标混合工作溶液,配制成系列质量浓度(0.01、0.02、0.05、0.1、0.2、0.5、1、2、5、10、20、50 μg/L)的基质匹配混合标准溶液。

### 1.3 样品前处理

对所有鱼类样品进行解剖和清洗,去除皮肤、头部、尾部、内脏及骨,对剩余组织进行匀浆处理,之后于-18 ℃条件下保存。

准确称取2.0 g上述匀浆后的样品,置于50 mL离心管中,加入20 μL内标混合工作溶液,混合均匀,随后加入5 mL超纯水和10 mL 2%甲酸乙腈,超声提取15 min;向上述样品中加入0.9 g NaCl和3.6 g无水MgSO_4_,涡旋振荡1 min,在3000 r/min下离心5 min;离心后取2 mL上清液至含有20 mg Fe_3_O_4_-TiO_2_、20 mg PSA和120 mg无水MgSO_4_的离心管中,充分混匀30 s后再置于磁分离架上进行分离;经3 s磁分离后,取1 mL上清液过0.22 μm有机滤膜,进行UHPLC-MS/MS分析。

为了控制样品前处理过程可能带来的外源性污染,实验过程中均避免使用聚四氟乙烯材质的器皿。

### 1.4 仪器分析条件

色谱柱:Luna Omega C18柱(100 mm×2.1 mm, 1.6 μm,美国Phenomenex公司);柱温箱温度:35 ℃。流动相:A相为10 mmol/L乙酸铵甲醇-水(1∶9, v/v), B相为10 mmol/L乙酸铵甲醇,流速0.25 mL/min。梯度洗脱程序:0~10 min, 10%B~100%B; 10~13 min, 100%B; 13~13.5 min, 100%B~10%B; 13.5~15 min, 10%B。总运行时间:15 min;进样体积:2 μL。

离子源:电喷雾电离(ESI)源;毛细管电压:4000 V;毛细管温度:300 ℃;加热块温度:400 ℃;脱溶剂管温度: 250 ℃。干燥气(氮气)、加热气(空气)、雾化气(氮气)的流速分别为10、10、3 L/min,其中氮气由氮气发生器提供,碰撞气为氩气。数据采集模式:多反应监测(multiple reaction monitoring, MRM)模式。13种PFASs的质谱分析参数如表1所示,其中每个离子对的驻留时间为2 ms。

**表1:** 13种PFASs和2种内标的质谱参数

No.	Compound	Molecular formula	Retention time/min	Precursor ion (m/z)	Product ions (m/z)	CEs/ eV	Internal standard
1	perfluorobutanesulfonic acid (PFBS)	C_4_HF_9_O_3_S	6.350	299	80^*^, 99	36, 36	^13^C_4_-PFOS
2	perfluorohexanoic acid (PFHxA)	C_6_HF_11_O_2_	7.285	313	269^*^, 119	4, 40	^13^C_2_-PFDA
3	perfluoroheptanoic acid (PFHpA)	C_7_HF_13_O_2_	8.098	363	319^*^, 169	6, 16	^13^C_2_-PFDA
4	perfluorohexanesulfonic acid (PFHxS)	C_6_HF_13_O_3_S	8.139	399	80^*^, 99	46, 39	^13^C_4_-PFOS
5	perfluoroheptanoic acid (PFHpS)	C_7_HF_15_O_3_S	8.679	449	80^*^, 99	50, 40	^13^C_4_-PFOS
6	perfluorooctanoic acid (PFOA)	C_8_HF_15_O_2_	8.694	413	369^*^, 169	6, 16	^13^C_2_-PFDA
7	perfluorooctane sulfonic acid (PFOS)	C_8_HF_17_O_3_S	9.129	499	99^*^	52	^13^C_4_-PFOS
8	chlorinated polyfluoroalkyl ether sulfonic acid (F-53B)	C_8_ClF_16_O_4_SK	9.397	531	351^*^	25	^13^C_4_-PFOS
9	perfluorodecanoic acid (PFDA)	C_10_HF_19_O_2_	9.558	513	469^*^, 219	8, 14	^13^C_2_-PFDA
10	perfluorodecane sulfonic acid (PFDS)	C_10_HF_21_O_3_S	9.856	599	80^*^, 99	60, 58	^13^C_4_-PFOS
11	perfluoroundecanoic acid (PFUnDA)	C_11_HF_21_O_2_	9.901	563	519^*^, 269	10, 18	^13^C_2_-PFDA
12	perfluorolauric acid (PFDoDA)	C_12_HF_23_O_2_	10.298	613	569^*^, 269	10, 18	^13^C_2_-PFDA
13	perfluorotridecanoic acid (PFTrDA)	C_13_HF_25_O_2_	10.461	663	619^*^, 269	10, 24	^13^C_2_-PFDA
14	sodium perfluoro-1-[1,2,3,4-^13^C_4_] octanesulfonate	-	9.130	502.9	80^*^, 99	55, 42	-
	(^13^C_4_-PFOS)						
15	perfluoro-n-[1,2-^13^C_2_] decanoic acid (^13^C_2_-PFDA)	-	9.559	514.9	470^*^, 219	10, 17	-

CEs: collision energies; * quantitative ion; -: no information.

## 2 结果与讨论

### 2.1 样品前处理条件的优化

#### 2.1.1 萃取溶剂类型

鱼类产品富含蛋白质和脂肪,并且不同PFASs的性质也各有差异,因此选择合适的萃取溶剂是提取PFASs和避免共萃取基质干扰的关键所在。乙腈是QuEChERS方法中常用的萃取溶剂,也是一种有效的蛋白质沉淀剂。PFASs的结构中含有羧基或磺酸基等端基,在酸性环境下,PFASs处于非解离状态,有利于进入有机相。因此,本研究通过在乙腈中加入不同体积分数的甲酸来考察萃取溶剂类型对13种PFASs回收率的影响。前处理条件优化过程选取鲈鱼作为样品基质,13种PFASs的添加水平均为100 μg/kg,每个条件下设定3个平行样品。实验分别考察了乙腈、1%甲酸乙腈、2%甲酸乙腈对13种PFASs回收率的影响。结果如图1所示,当萃取溶剂为乙腈时,除PFOA(回收率为65.1%)外,其余12种PFASs的回收率为71.8%~88.4%;使用1%甲酸乙腈萃取目标物时,与乙腈相比,除PFOA外,其余12种PFASs的回收率均呈现下降趋势,且仅有4种PFASs的回收率在70%以上;当萃取溶剂为2%甲酸乙腈时,13种PFASs的回收率为72.3%~107%。因此,最终选择2%甲酸乙腈作为萃取溶剂。

**图1:**
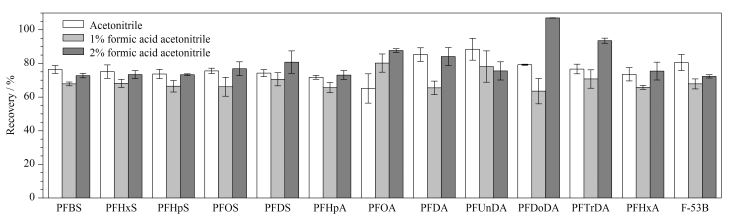
不同萃取溶剂对13种PFASs回收率的影响(*n*=3)

#### 2.1.2 Fe_3_O_4_-TiO_2_和PSA用量

选用Fe_3_O_4_-TiO_2_和PSA作为净化吸附剂,其中Fe_3_O_4_-TiO_2_可以通过范德华力、静电相互作用以及氢键等作用力来吸附基质溶液中的蛋白质等杂质^[22]^; PSA作为弱阴离子交换吸附剂,可与脂肪酸等分子中的羟基形成非共价键,从而达到净化作用^[23]^。待净化过程完成后,在外置磁场下,Fe_3_O_4_-TiO_2_能够实现固相与液相的快速分离^[24,25]^。样品经2%甲酸乙腈萃取后,加入不同质量(10、20、30、40、50 mg)的Fe_3_O_4_-TiO_2_、30 mg PSA和120 mg无水MgSO_4_,考察Fe_3_O_4_-TiO_2_用量对13种PFASs回收率的影响。如图2所示,当Fe_3_O_4_-TiO_2_用量为10 mg时,PFDS、PFUnDA和PFHxA的回收率均小于70%;当Fe_3_O_4_-TiO_2_的用量为20 mg时,13种PFASs的回收率为70.7%~83.8%;当Fe_3_O_4_-TiO_2_用量为30、40和50 mg时,多数PFASs的回收率为60%~70%,这可能是因为过量的Fe_3_O_4_-TiO_2_会对目标物产生吸附作用。因此,确定Fe_3_O_4_-TiO_2_的用量为20 mg。

**图2:**
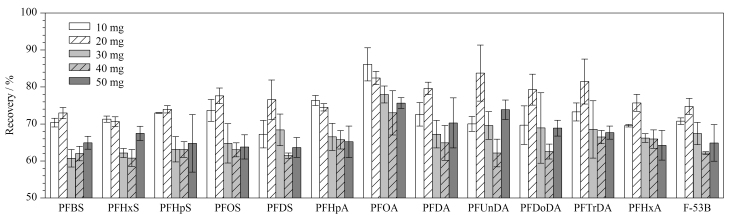
Fe_3_O_4_-TiO_2_用量对13种PFASs回收率的影响(*n*=3)

样品经2%甲酸乙腈萃取后,加入不同质量(10、20、30、40和50 mg)的PSA、20 mg Fe_3_O_4_-TiO_2_及120 mg无水MgSO_4_,考察PSA用量对13种PFASs回收率的影响。结果如图3所示,当PSA用量分别为10、20、30、40、50 mg时,回收率为70%~120%的PFASs分别有11、13、12、13和8种;当PSA用量为40 mg时,13种PFASs的回收率为70.8%~98.6%,其中有10种PFASs的回收率为70%~80%;当PSA用量为20 mg时,13种PFASs的回收率为77.0%~106%,且相对标准偏差(RSD)均小于6.1%,因此确定PSA用量为20 mg。在最优条件下,13种PFASs的总离子流色谱图见图4。

**图3:**
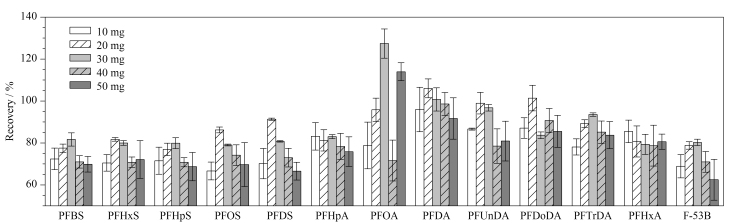
PSA用量对13种PFASs回收率的影响(*n*=3)

**图4:**
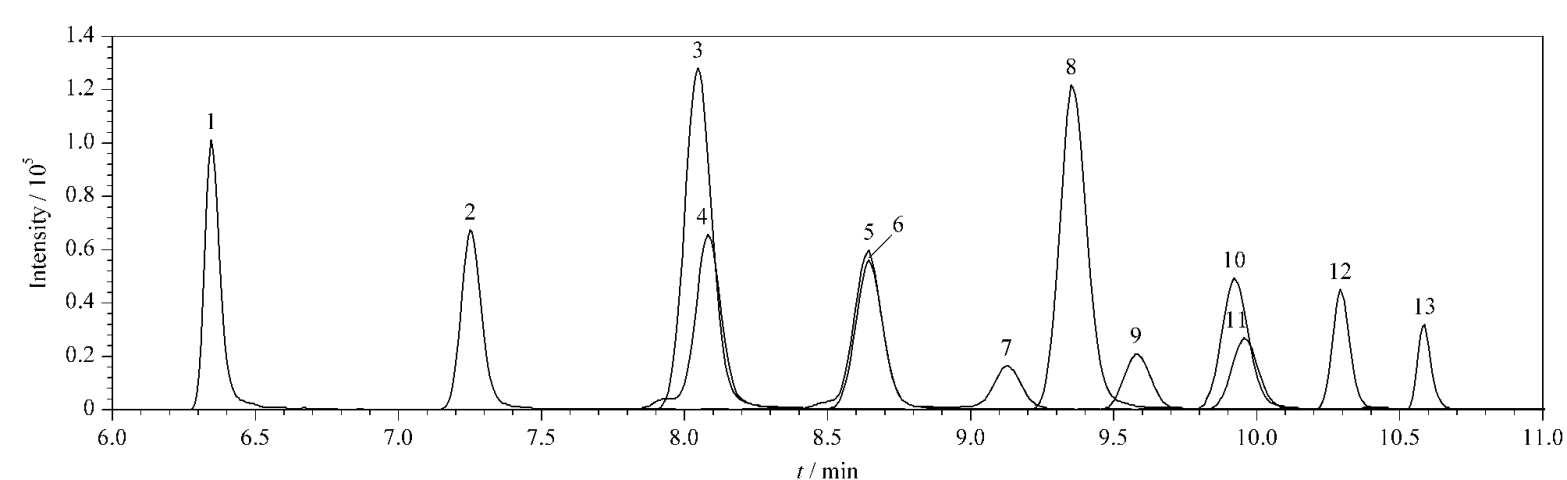
最优条件下13种PFASs(100 μg/kg)的总离子流色谱图

### 2.2 线性关系、检出限和定量限

本研究采用内标法对13种PFASs进行定量分析。配制系列质量浓度的基质匹配混合标准溶液,以目标分析物与相应内标的平均峰面积之比为纵坐标(*Y*)、质量浓度之比为横坐标(*X*),绘制标准曲线,并通过线性相关系数(*R*)来验证线性关系。分别以3倍和10倍信噪比(*S/N*)所对应的PFASs质量浓度作为检出限(LOD)和定量限(LOQ)。结果如表2所示,13种PFASs在0.01~50 μg/L内均具有良好的线性关系,*R*为0.9973~0.9999, LOD为0.001~0.023 μg/L, LOQ为0.003~0.078 μg/L,说明本方法能够满足13种PFASs的测定需求。

**表2:** 13种PFASs的线性方程、相关系数、检出限、定量限及基质效应

Compound	Linear equation	R	LOD/ (μg/L)	LOQ/ (μg/L)	ME
PFBS	Y=1.21X+0.23	0.9995	0.008	0.027	0.89
PFHxA	Y=0.52X+0.21	0.9993	0.005	0.018	0.76
PFHpA	Y=0.98X+0.28	0.9981	0.009	0.030	0.80
PFHxS	Y=1.27X+0.15	0.9997	0.013	0.042	0.99
PFHpS	Y=1.08X+0.07	0.9994	0.023	0.078	1.02
PFOA	Y=0.22X+1.15	0.9973	0.003	0.009	0.86
PFOS	Y=0.50X+0.18	0.9992	0.004	0.014	1.02
F-53B	Y=3.35X+0.24	0.9999	0.001	0.003	1.10
PFDA	Y=0.18X+0.11	0.9981	0.001	0.003	0.88
PFDS	Y=0.93X+0.02	0.9999	0.021	0.068	1.10
PFUnDA	Y=0.18X+0.11	0.9999	0.001	0.003	0.79
PFDoDA	Y=0.24X-0.01	0.9998	0.001	0.003	0.92
PFTrDA	Y=0.15X+0.01	0.9987	0.001	0.003	0.98

*Y*: peak area ratio of target analyte to internal standard; *X*: mass concentration ratio of target analyte to internal standard. Linear ranges of the 13 PFASs were 0.01-50 μg/L.

### 2.3 基质效应

本实验通过计算基质匹配混合标准曲线与溶剂混合标准曲线的斜率之比来评价13种PFASs的基质效应(ME)。当ME为0.8~1.2时,说明基质效应不明显;当ME为0.5~0.8或1.2~1.5时,说明存在中等强度的基质效应;当ME<0.5或>1.5时,则表示基质效应强烈^[26]^。实验结果表明,13种PFASs的ME为0.76~1.10(表2),说明本方法的基质效应可以忽略。

### 2.4 回收率和精密度

在空白鲈鱼样品中添加低、中、高3个水平(0.5、10、100 μg/kg)的标准品,在优化的条件下进行加标回收试验,进样分析后计算13种PFASs的回收率;每个加标水平在1 d内平行测定3次,计算日内精密度(intra-day RSD, *n*=3);在第1、3、5 d分别进行测定,计算日间精密度(inter-day RSD, *n*=3)。结果表明,在3个加标水平下,13种PFASs的回收率为78.1%~118%,日内RSD和日间RSD分别为0.2%~11.1%和0.8%~8.7%(表3)。上述结果说明,本文所建立方法具有良好的回收率和精密度。

**表3:** 13种PFASs在3个加标水平下的回收率及日内、日间精密度(*n*=3)

Compound	Recoveries/%				Intra-day RSDs/%				Inter-day RSDs/%		
	0.5 μg/kg	10 μg/kg	100 μg/kg		0.5 μg/kg	10 μg/kg	100 μg/kg		0.5 μg/kg	10 μg/kg	100 μg/kg
PFBS	99.9	93.9	84.8		6.1	0.2	7.5		5.0	4.8	8.7
PFHxA	86.6	84.6	84.3		0.7	1.4	6.0		6.3	1.8	6.3
PFHpA	99.1	95.7	87.9		0.4	0.5	3.1		7.3	3.5	5.8
PFHxS	94.1	93.9	87.1		11.1	1.3	4.0		5.3	5.3	7.1
PFHpS	81.3	98.2	90.2		7.8	2.9	4.4		5.1	0.8	6.6
PFOA	88.0	104	78.1		7.6	2.1	5.4		3.6	2.2	4.5
PFOS	98.9	97.0	86.7		5.8	0.2	4.4		5.0	2.1	7.6
F-53B	82.1	94.8	88.5		7.4	2.6	3.0		4.7	1.8	7.0
PFDA	118	103	95.7		6.2	1.0	5.0		7.0	6.7	6.5
PFDS	108	94.6	88.2		1.5	0.8	6.0		4.0	2.0	5.0
PFUnDA	115	101	89.9		7.6	6.3	4.7		5.6	4.3	6.8
PFDoDA	85.2	109	98.1		9.3	2.5	8.8		4.6	3.4	6.8
PFTrDA	89.0	108	106		6.1	5.2	9.9		4.3	4.5	4.5

### 2.5 实际样品分析

在浙江省不同养殖基地采集11个鱼样品(包含4个鲤鱼、3个鲈鱼、2个白条鱼、1个鲫鱼和1个大黄鱼),并利用本文建立方法进行检测,以验证该方法的实用性。当测定结果低于LOQ时,认为样品无检出。结果如表4所示,11个鱼类样品均有PFASs检出,检出的PFASs分别为PFOS、PFUnDA、PFOA、PFDoDA和PFTrDA;其中,PFOS的检出率(100%)和检出总含量(1.728 μg/kg)最高,其次分别为PFUnDA(检出总含量1.208 μg/kg)、PFOA(检出总含量0.931 μg/kg)、PFDoDA(检出总含量0.680 μg/kg)和PFTrDA(检出总含量0.327 μg/kg),这与文献[29]报道结果基本一致;并且,除PFOS外,其余4种检出的PFASs均属于长链羧酸类PFASs。由上述结果推测,鱼类样品中的主要PFASs污染因子为PFOS及长链羧酸类PFASs。根据文献[30]报道,基于鱼体的代谢机制,长链羧酸类PFASs及PFOS在鱼类样品中具有很强的生物累积和生物放大能力。此外,对长链羧酸类PFASs的检出原因进行分析,由于长链羧酸类PFASs具有较高的亲脂性,其在生物体内累积的可能性更高,同时PFASs在生物体内的富集作用会随其链长的增加而增大。上述结果说明,本文所建立方法在实际鱼类样品检测中具有较大的应用潜力。

**表4:** 实际样品的测定结果

No.	Sample type	PFOA	PFUnDA	PFDoDA	PFTrDA	PFOS
1	cyprinoid	0.056	0.038	ND	ND	0.227
2	cyprinoid	0.162	0.030	ND	ND	0.055
3	cyprinoid	0.144	0.283	0.091	0.100	0.343
4	cyprinoid	0.087	0.026	ND	ND	0.042
5	Lateolabrax japonicus	0.076	ND	ND	ND	0.065
6	Lateolabrax japonicus	ND	ND	ND	0.015	0.053
7	Lateolabrax japonicus	0.034	0.132	0.038	0.272	0.169
8	Hemiculter leucisculus	0.077	0.040	ND	0.018	0.071
9	Hemiculter leucisculus	0.076	0.032	ND	0.020	0.075
10	crucian	0.121	0.240	0.052	0.025	0.354
11	Larimichthys crocea	0.098	0.387	0.146	0.230	0.274

ND: not detected.

### 2.6 方法比对

将本文建立方法与其他文献报道方法进行比较,结果如表5所示。与单独的SPE^[27]^和QuEChERS方法^[28]^相比,本方法的单个样品预处理时间仅需25 min,净化吸附剂用量少,检出限低且回收率高,具有环保、经济、操作简便等优点。

**表5:** 本方法与其他文献方法的比较

Preparation method	Adsorbent amounts	Pretreatment time/min	LODs	Recoveries/ %	Ref.
SPE	150 mg WAX and Envi-Carb	120	0.2-3.1 μg/kg	67-126	[27]
QuEChERS	100 mg PSA, 40 mg C18, 20 mg GCB, 2 g anhydrous MgSO_4_	90	0.01-1.0 μg/kg	61-117	[28]
Magnetic d-SPE	20 mg Fe_3_O_4_-TiO_2_, 20 mg PSA, 120 mg anhydrous MgSO_4_	25	0.001-0.023 μg/L	78.1-118	this work

WAX: weak anion-exchange; Envi-Carb: graphitized carbon black filler; d-SPE: dispersive-solid phase extraction; GCB: graphene carbon black.

## 3 结论

本研究在传统QuEChERS的基础上,将磁性功能材料作为d-SPE净化吸附剂,建立了鱼类产品中13种PFASs的UHPLC-MS/MS分析方法。实验系统考察了萃取溶剂类型、净化吸附剂用量对PFASs回收率的影响,并获得了最佳样品前处理条件。所建方法灵敏、简便且适用性广,能够满足实际鱼类样品的分析要求,为进一步开展鱼类产品中PFASs污染物监测、阐明和评估复杂基质中PFASs的风险提供了一种新型、高效的检测手段。
